# Behavior of Aqueous Medicated Inks on Porous Tablet Surfaces

**DOI:** 10.3390/pharmaceutics17070908

**Published:** 2025-07-14

**Authors:** Krisztina Ludasi, Anna Sass, Katalin Kristó, András Kelemen, Klára Pintye-Hódi, Tamás Sovány

**Affiliations:** 1 Institute of Pharmaceutical Technology and Regulatory Affairs, University of Szeged, Eötvös u 6., H-6720 Szeged, Hungary; 2 Department of Technical Informatics, University of Szeged, Tisza Lajos krt. 103., H-6720 Szeged, Hungary

**Keywords:** inkjet printing, porous substrates, tablets, labeling, personalized medicine

## Abstract

**Background/Objectives:** Although technology has progressed and novel dosage forms have been developed, tablets are still the most used form of medication. However, the present manufacturing methods of these oral solid dosage forms offer limited capacity for personalized treatment and adaptable dosing. Personalized therapy, with a few exceptions, is not yet a part of routine clinical practice. Drug printing could be a possible approach to increase the use of personalized therapy. The aim of this work was to investigate the role of surface tension and the viscosity of inks in the formation of the printing pattern and to investigate how the porosity of substrate tablets influences the behavior of inks on the surface. **Methods:** Spray-dried mannitol served as a binder and filler, while magnesium stearate functioned as a lubricant in the preparation of substrate tablets. Brilliant Blue dye was a model “drug”. The ink formulation was applied to the substrates in three varying quantities. **Results:** Increasing the viscosity enhanced the drug content, potentially improving printing speed and pattern accuracy. However, it negatively impacted the dosing accuracy due to nozzle clogging and prolonged drying time. Viscosity had a significantly higher impact on the ink behavior than surface tension. Lowering the surface tension improved the dosing accuracy and reduced the drying time but resulted in smaller drop sizes and decreases in pattern accuracy. Reducing the substrate porosity led to longer drying times and diminished pattern accuracy. **Conclusions:** A target surface tension of around 30 mN/m is suggested for inkjet printing. It is necessary to further investigate the applicability of the technology with solutions of inks with high viscosity and low surface tension, including the API.

## 1. Introduction

Despite technological advances and the emergence of new dosage forms, tablets and capsules remain the most used types of medicines today. This is mainly due to their advantages, such as dosing accuracy, chemical and microbial stability, the possibility of controlled release, and their ease of administration [1,2]. Nevertheless, the currently used manufacturing methods of these oral solid dosage forms have limited potential to provide personalized medicines and flexible dosing. Commercially available medicines are usually available in only two or three different strengths, which do not adequately meet the needs of pediatric patients and elderly people of different ages [3,4,5,6].

The challenges of “standardization” and “individualization” have always been typical features of health services. The best possible care in terms of individuality is an ethical imperative of medicine to which all patients have a right. However, in terms of standardization, all available treatments are based on guidelines from large, multicentric studies involving thousands of patients. The concept of “one drug for all patients with the same disease” is not appropriate, as it has been observed that groups of patients may respond differently to a particular drug, for example, because of the differences in enzyme systems involved in drug metabolism [7,8], so a more individualized approach is needed [9,10]. The mapping of the entire human genome has enabled the emergence of new disciplines in the 21st century, e.g., pharmacogenetics and pharmacogenomics, which have provided key foundations for personalized therapy [11,12,13].

Personalized medicine aims to provide personalized prevention and treatment strategies for specific groups of individuals [14,15] and is promoted in the hope that new technical options for the estimation of health risks, monitoring disease progression, and predicting response to therapy would enable this personalized, preventive approach to care [16,17]. Combining therapy with drug-related diagnostics can provide better recovery parameters for new products, as well as improve the safety profile or efficacy of older drugs [18].

Due to the limited number of commercially available dosing strengths, the splitting of scored tablets can be an approach of individualized therapy, enabling further dose adjustment. Commonly available scored tablets can be split into halves or quarters, but there is a risk in segmenting tablets, as splitting may cause dose fluctuation, and numerous patients find it challenging to break up unbreakable preparations, or some patients break pills that should be taken intact [19,20].

Inkjet printing (IJP) and 3D printing have proved to be more suitable techniques for realizing personalized, low-cost therapies, providing higher reproducibility and better dose accuracy [1,21,22,23,24], but are more time-consuming procedures than tableting. Sustainability, patentability, and lower-cost manufacturing are benefits that can also justify an interest in printing [25]. The example of prednisolone demonstrates the potency of these techniques to enable dose flexibility, as the currently availably doses cannot fulfill the need for carefully controlled and variable doses during treatment. With IJP, the number of doses can be increased, which could lead to a higher level of personalization and increased patient cooperation [24]. IJP technology is represented at important stages of the life cycle of medicines, from research and development to production, labeling, and packaging of therapeutic formulations. Due to its flexibility, this printing process can be easily integrated into individual processes, and its advantages, such as controlled, reproducible, and optimizable ink droplet sizes; its small space requirement; and online control, allow the method to be used in high-throughput discovery studies [26,27].

IJ printers eject liquid droplets (e.g., API solution) from a nozzle, which travel a few mm through the air to be deposited on the surface of a substrate in a noncontact mode. Based on how the ink droplets are formed, two methods are known: continuous (CIJP) and drop-on-demand (DoD) IJP. CIJP uses a continuous pressure flow in conjunction with a valve that opens and closes to release the stream droplets, while DoD techniques shoot out small amounts of liquid from the printhead only when a drop is required in response to an electrical signal. It uses two types of printheads: thermal and piezoelectric. Piezo actuation uses a volume change to induce the pressure required to eject the droplets, while a thermal inkjet (bubble jet) creates gas pockets by rapidly heating samples to the required pressure needed to eject the droplets [27]. Both types of operation have advantages and disadvantages. Piezoelectric printheads can handle a wider range of liquids than thermal printheads, which are limited to liquids that evaporate well, although the production of these can be easier and cheaper. DoD printing can use small volumes of liquid and so is used in most research applications in the pharmaceutical field in contrast with CIJP, which requires a significant recirculation volume. Typical droplet diameters in DoD printing are between 10 and 50 μm, corresponding to a droplet volume of 1–70 pL [24]. IJP technology is being promoted for personalized medicine because, although it is slower than flexographic printing, it can be used to deliver API-containing inks precisely and in small volumes, making it highly suitable for the individualized delivery of pharmaceuticals with small therapeutic indexes [28].

Nevertheless, IJP technology faces many challenges in pharmaceutical applications, as the development of drug-based inks is complicated due to the necessity to ensure drug compatibility, stability, and uniform distribution. Optimization of the formulation, including the selection of solvents, stabilizers, and other additives, is extremely complex [29]. The drug must be carefully dissolved, dispersed, or encapsulated in an appropriate solvent or carrier to produce a homogeneous and printable ink formulation. Each type of pharma-ink requires specific excipients to optimize the ink properties and drug delivery characteristics [30]. Furthermore, the printing itself must be extremely precise, as the accurate dosing of drugs is crucial, but technical obstacles can occur during printing, such as clogged nozzles or a lack of uniform ink distribution [31]. In the field of pharmaceutical printing, the development of inks for pharmaceutical applications is more based on trial and error, but recently, attempts were made to use more advanced optimization methods [32]. In the study of Schulz et al., the Z-number was highlighted as a key parameter to judge the printability of an ink. They investigated the limits of printability for solvent-based inks using a Spectra S Class SL-128 piezo printhead. They found that the printability of inks can be more effectively judged by capillary and Weber numbers. The jettable window enables formulation scientists to identify if an ink will be still jettable after a composition change based on its change in viscosity, density, or surface tension. These parameters could be used to calculate whether an ink is jettable or not based on the jettable window, without the need for experimental trials in the future [33].

The following pharmaceutical products are under development in combination with IJP as delivery systems: orodispersible films, mucoadhesive buccal films, 3D-printed tablets, capsules, bioadhesive films, microneedles and patches, contact lenses, etc. [34]. However, most of the published studies used various kind of films.

In a study on theophylline, a drug with a narrow therapeutic index, researchers successfully applied it to a film of Tamarindus indica Linn. seed meal softened with sorbitol, with high precision and accuracy and that released in 5 min during the dissolution phase [35]. In another study, Eleftheriadis et al. prepared a hydroxypropyl methylcellulose-based buccal film for the co-release of ketoprofen and lidocaine HCl. Unidirectional release was achieved by incorporating an ethyl cellulose-based backing layer. Lidocaine HCl, in combination with permeation-enhancing l-menthol, was loaded to the film via inkjet printing [36]. In another study, sodium picosulfate containing aqueous ink was IJ-printed (by a piezoelectric DoD printer) onto classically used edible substrate films such as potato starch, rice paper, icing sheet, pure HPMC films, chitosan films, cast films with polymers, and plasticizers used in pharmaceutical products to study ink–substrate interactions [37], and it was concluded that most of these substrates can prevent crystallization and enable fast release.

Sandler et al. used three different print substrates—uncoated paper, coated paper, and polyethylene terephthalate (PET) film—which were IJ-printed with ink solutions containing paracetamol, caffeine, and theophylline dissolved in a mixture of propylene glycol and purified water. Substrate properties such as the porosity, surface area, and hydrophilicity significantly affected the drug release rate. For example, highly porous substrates with a high surface area provided a larger area for drug diffusion, resulting in faster drug release. In contrast, less porous substrates were found to impede drug diffusion, leading to slower release [38]. In another study, Genina et al. evaluated the behavior of drug-containing inks on different substrates (PET film, HPC film, and icing sheets), focusing on wettability, morphology, and crystallinity. Measurements of contact angles revealed better spreading of ink with lower surface tension on PET and HPC films, where ink droplets remained visible for several minutes, while they were rapidly absorbed on porous icing sheets. The degree of recrystallization and crystal sizes depended on the substrate and dose. This confirms that substrate properties, such as thickness and wettability, significantly influence ink behavior and drug morphology. Overall, the study highlights key factors affecting the dosing accuracy and printing outcomes in pharmaceutical inkjet applications [39].

However, the diversity of substrates can pose problems of incompatibility, and optimization of the physical and chemical properties can also be challenging. In addition to substrate stability, the effective absorption of drugs is also important. Therefore, much remains to be explored in this area.

This work focuses on the investigation of how the physicochemical characteristics (e.g., the surface tension and viscosity) of an applied medicated ink influence the printing and dosing accuracy, as well as its behavior on a porous tablet substrate, which is a field that is currently underexplored in the literature despite its importance not only for personalized medicine but also for anti-counterfeiting purposes [40]. Recently, many pharmaceutical companies have adopted inkjet printing to label tablets due to its low cost and versatility. Although the printed characters on tablets are intended to be identical, microscopic variations caused by surface irregularities and tablet orientation during printing result in unique, unclonable patterns. Ishiyama et al. proposed the use of these subtle differences—referred as “fingerprints”—for the individual identification and authentication of tablets [41].

Also, in the study by Trenfield et al., 2D and 3D printing technologies were combined in drug formulation. The authors used 2D printing to apply codes and data matrices onto 3D-printed tablets, which could be scanned with a smartphone to retrieve information about the medication, the patient, and the prescriber. Additionally, as part of the anti-counterfeit strategy, a unique combination of inks was printed onto the tablet surface using a 2D printer, enabling detection via Raman spectroscopy [42].

Building on the previous developments carried out at our institute, one of the planned initiatives is to continue the development of anti-counterfeiting technologies for pharmaceutical tablets, which, until now, have been implemented using laser ablation [43,44,45]. Once IJP technology is successfully developed, it could serve as a new platform for these advancements.

## 2. Materials and Methods

### 2.1. Materials

Spray-dried mannitol (Pearlitol SD 200, Roquette Pharma, Lestrem, France) was used as binder/filler, and magnesium stearate (Molar Chemicals Kft., Halásztelek, Hungary) was used as lubricant for making of substrate tablets. Purified water was used as solvent, while polyvidone (PVP) (Kollidon 25, BASF ChemTrade GmbH, Burgberheim, Germany) and Polysorbate 20 (PS) (Merck KGaA, Darmstadt, Germany) were used as excipients to set the physicochemical properties (viscosity and surface tension, respectively) of the inks. Brilliant Blue FCF dye (BASF ChemTrade GmbH, Burgberhaim, Germany) was used as model “drug”. All materials were used as received, and their quality meets the requirements of the Ph. Eur.

### 2.2. Methods

#### 2.2.1. Preparation of Tablets

Pearlitol SD 200 (99% *w*/*w*) and the lubricant magnesium stearate (1% *w*/*w*) were homogenized by a Turbula mixer (Willy A. Bachofen Maschienenfabrik, Muttenz, Switzerland) at 50 rpm for 2 min. Round tablets, with a mass of 0.2 g and 13 mm diameter, were compressed from the homogenous powder mixture using a hydraulic press (Specac Inc, Orpington, UK) at 3, 4, and 5 ton-force (corresponding to 165, 220, and 275 MPa compression pressure, respectively) to achieve different porosities. For better bond consolidation, 30 s dwell time was used at the maximum force.

#### 2.2.2. Determination of Table Characteristics

The height and diameter of the tablets were measured with a screw micrometer (Mitutoyo, Tokyo, Japan), while tablet mass was measured with an analytical balance (Startorius AG, Göttingen, Germany). The apparent density of the tablets was calculated with the following Equation (1):

(1) $$\rho_{a}=\frac{m}{{\left(\frac{d}{2}\right)}^{2}\pi h}$$

where *ρ_a_* is the apparent density, *m* is the mass, *d* is the diameter, and *h* is the height of the tablets. The true density of tablets was determined with a helium pycnometer (Quantachrome Instruments, Boynton Beach, FL, USA). The porosity was then calculated using Equation (2):

(2) $$\varepsilon =\left(1-\frac{\rho_{a}}{\rho_{t}}\right)\times 100$$

where ε is the porosity, *ρ_a_* is the apparent density, and *ρ_t_* is the true density of tablets.

#### 2.2.3. Preparation of the Inks

Required amounts of PS and PVP were dissolved in approx. 30 mL of purified water according to a 3^2^ full factorial experimental design (Table 1), followed by the dissolution of the model material Brillant Blue FCF dye in the amount of 0.016 g/mL, which ensured the traceability of the ink penetration and helped to follow the ink distribution on the substrate. The amount of solution was finally set to 40 g with purified water. Solutions were stored in a refrigerator until further use.

#### 2.2.4. Printing

**Figure 1:**
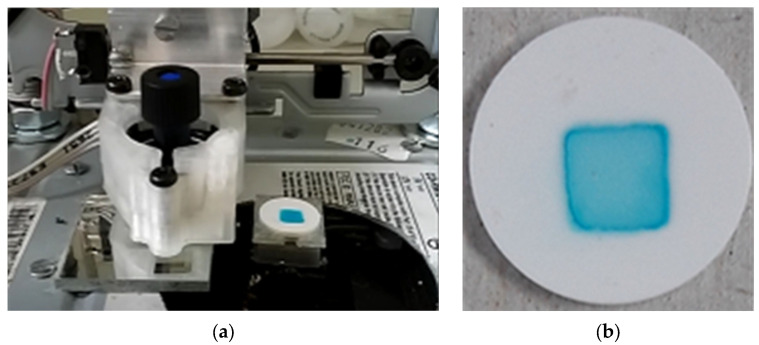
(**a**) Photo of the applied UniPharmPrint inkjet printer; (**b**) 20 × 20 pixel square-like printing pattern on substrate compressed at 5 t.

The printing experiments were conducted with a self-developed piezoelectric inkjet printing apparatus, UniPharmPrint (Figure 1a).

The device is operated by computer software developed for it. The print pattern can be adjusted to the right position on the substrate by the setting of vertical and horizontal displacement of the printhead. The ink solutions were filtered through a 0.22 µm pore size membrane filter (CHROMAFIL^®^ Xtra PVDF, 0.22 µm, Macherey-Nagel GmbH & Co. KG, Dueren, Germany) prior to filling into the printer tank. Printing was performed through a single nozzle, using 110 dpi resolution and resulting in a square of 20 × 20 pixels (Figure 1b). Each ink composition was printed on substrates using three different amounts (e.g., 10, 50, and 90 drops/pixel). The pattern accuracy was then evaluated later qualitatively and quantitatively. The quantification of pattern accuracy was done with ImageJ 1.54g software (National Institute of Health, Bethesda, MD, USA) through the analysis of the area and solidity of the printed spot. The spot solidity can be calculated with the following Equation (3):

(3) $$Solidity=\frac{Spot\;area}{Convex\;spot\;area}$$

#### 2.2.5. Determination of the Surface Tension of Inks and Wettability of Substrates

Prior to performing the printing experiments, preformulation studies were done to determine the surface tension of the inks and their spreading characteristics (e.g., the contact angle of the droplet) on the substrate surface. Measurements were performed using a DataPhysics OCA 20 (DataPhysics GmbH, Filderstadt, Germany) optical contact angle tester. The surface tension of the inks was determined using pendant drop method, and the calculation was made by the SCA 20 software of the device (DataPhysics GmbH, Filderstadt, Germany) based on the Laplace–Young Equation (4).

(4) $$∆p=\sigma \left(\frac{1}{R_{1}}+\frac{1}{R_{2}}\right)$$

where ∆*p* is interfacial pressure difference, *σ* is the interfacial tension, and *R*_1_ and *R*_2_ are the radii of the curvature of the surface.

The relationship characterizes the pressure difference that determines the shape of the droplet, which is a result of the gravitational force and the surface tension that tend to contract the surface of the droplet. For each solution, 10 droplets were generated at the end of a 40 μL Hamilton pipette and were tested immediately prior to dropping, with 5 measurements per droplet.

The wettability of the substrates was calculated by the software using sessile drop method. A 10-10 drop of each ink solution was dropped on the surface of tablets prepared at different compression forces. The changes in contact angle and droplet volume were detected for 40 s, performing circle fitting in every second.

#### 2.2.6. Determination of Drying Time

Printed samples were dried under ambient conditions (25 ± 2° C and 50 ± 5% rH) during the printing stage. Drying time was determined with stopwatch (VWR Hungary Ltd., Debrecen, Hungary) by visual observation. The time when the glossy surface became matte due to the disappearance of visible droplets was considered as the drying time.

#### 2.2.7. Determination of Drug Content

The tablets previously used to determine the drying time were further tested to measure the amount of brilliant blue dye printed on them. Samples were dissolved in 10 mL of purified water at room temperature. Solutions were then filtered through a 0.22 µm pore size membrane filter (CHROMAFIL^®^ Xtra PVDF, 0,22 µm, Macherey-Nagel GmbH & Co. KG, Dueren, Germany), and the concentration was determined at the absorption maximum of the dye (630 nm) using a Genesys 10 S UV-VIS spectrophotometer (ThermoScientific Inc., Waltham, MA, USA).

#### 2.2.8. Risk Assessment

**Figure 2:**
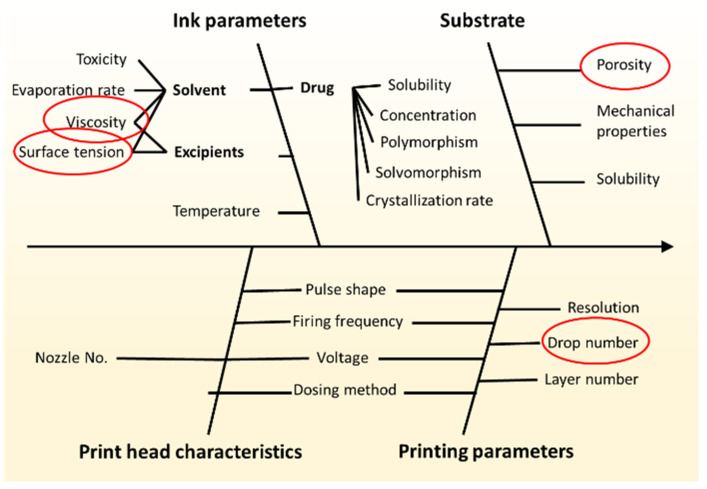
Ishikawa diagram of critical material attributes (CMAs) and critical process parameters (CPPs) for determining the quality of printed dosage forms.

Applying Quality by Design principles is a key issue in recent drug development. Risk assessment is one of the key features of the QbD concept, applying various quality tools that help to improve the quality of products and processes. In this research, an Ishikawa fishbone diagram (Figure 2) was created to summarize cause–effect connection between potential formulation, process parameters, and critical quality attributes (CQAs) of the product. Not only the main causes but also more detailed factors may be identified [46].

#### 2.2.9. Design of Experiments (DoE)

To explore the correlation between the controlled factors (ink composition, print settings, and substrate properties) and the printing result, a full factorial experimental design with response surface methodology, a widely used method to describe systems with multiple parameters in drug formulation studies, was applied using Tibco Statistica v.14.0.1.25 (Tibco Software Inc., Palo Alto, CA, USA). This method helps to find the correlations between the independent (critical material attributes (CMAs) or critical process parameters (CPPs)) and dependent variables (CQAs) under investigation by varying the values of several factors simultaneously, thus providing fast and complex information about the system of interest. For detailed description of the DoE methodology, please see the Supplementary Materials.

In the present paper, API content (y_1_), dosing accuracy (y_2_), drying time (y_3_), and two parameters of spot area (y_4_) and spot solidity (y_5_) describing the pattern accuracy were investigated as CQAs. The y_1_, y_2,_ y_4_, and y_5_ were investigated according to a 3^3^ design, while y_3_ was investigated according to a 3^4^ full factorial design. The corresponding independent variables (x_1_–x_4_) and their levels are shown in Table 2 and Table 3, respectively. The selection of factor levels was based on the results of the risk assessment. The ranges of the PVP and PS contents were determined to cover the widest possible range of viscosity and surface tension which can be handled by the applied printhead, with note that PS content fulfills the criteria for factors if it is interpreted on a logarithmic scale. The compression force was selected to cover a commonly used range by the pharmaceutical industry and to ensure different textures and porosities for the substrates.

The 4th CQA of the precision of the printed pattern was also examined as a function of these four variables, but as this could not be quantified, the findings on the correlations are based on visual inspection only.

## 3. Results and Discussion

### 3.1. Substrate Properties

The main characteristics of the tablets that served as substrates for printing are displayed in Table 4.

It is clearly visible that, as was expected, the porosity of the substrates decreased in a nonlinear way with increasing compression pressure, where an approx. 5% decrease can considerably influence the spreading and permeation of the ink into the deeper layers.

### 3.2. Preformulation Studies

The selection of a suitable solvent for the preparation of medicated inks can be challenging, as it should fulfill the requirements of both printability and biocompatibility. From the aspect of biocompatibility, purified water is definitely the best choice, but, due to its relatively high surface tension, its use may be challenging with respect to printing and drying. Polysorbate 20 is a well-known nonionic surfactant, with a high HLB value of 16.7. This enables the modification of the surface tension of water over a wide range without the risk of micelle formation, which is crucial for obtaining the required printability of the formulated ink. Moreover, it has better biocompatibility than SDS, SDBS, or other ionic surfactants with similarly high HLB values. Table 5 shows the surface tension values and their standard deviations, which were calculated using the SCA20 software based on the Laplace–Young equation.

It can be stated based on the measurements that the values of the surface tension values of all solutions were lower compared to the surface tension of the solvent, purified water, of 72 mN/m (at 25 °C). A systematic decrease was observed with increasing PS content for a given PVP concentration. In addition to the surface-active material, the increase in PVP content also reduced the surface tension, and the combined presence of the two components exhibited a synergistic effect. Based on the literature data, a surface tension of about 30 mN/m is suggested as the target value for inks suitable for printing [47]. Nevertheless, it should be noted that the specific value is always influenced by the specificity and physicochemical properties of the API used in the ink, as well as by the requirements of the actual printhead.

For the viscosity of the inks, the literature values were considered and were found to be 1, 2, and 3.5 mPas for samples containing 0, 5, and 5% PVP, respectively.

**Figure 3:**
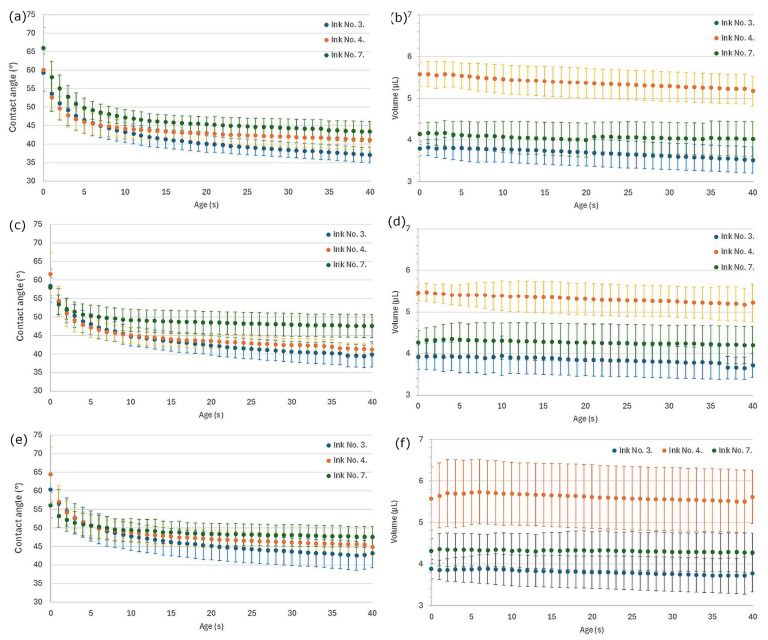
Behavior of droplets on substrate surfaces: (**a**) change in contact angle for substrate compressed with 165 MPa pressure, (**b**) change in volume for substrate compressed with 165 MPa pressure, (**c**) change in contact angle for substrate compressed with 220 MPa pressure, (**d**) change in volume for substrate compressed with 220 MPa pressure, (**e**) change in contact angle for substrate compressed with 275 MPa pressure, (**f**) change in volume for substrate compressed with 275 MPa pressure.

The values of the contact angle and volume of the ink droplet on the substrates were also monitored and then plotted as a function of time (Figure 3). As can be seen, the contact angle increased in order of ink 3, 4, and 7, which had viscosity values of 1, 2, and 3.5 mPas, respectively, which indicates that the increasing viscosity prevented spreading and helped to localize the droplet after printing. In the case of drop volume, a higher volume was detected in the case of ink 4, which had the highest surface tension (Table 5)—the result indicates that this parameter has a more considerable effect on drop size than viscosity. Observing the same solutions on different substrates, it was found that a smaller drop volume was observed in the case of the substrate compressed at 165 MPa, which further decreased with time, indicating considerable penetration of the ink into the substrate due to its higher porosity. In such cases, the decreasing contact angle may be connected more to penetration than to spreading.

### 3.3. API Content and Printing Accuracy

The Brilliant Blue dye applied to each tablet is shown in Table 6. There were significant differences in the values obtained at the same drop number, depending on the viscosity and surface tension of the ink, which were in good accordance with the results of the preformulation tests. After statistical evaluation, the following second-order polynomial was obtained Equation (5). For better prediction performance, neglectable factors were deleted from the equation to obtain the best fit, which was set by maximizing the adjR^2^ value. Those values with significant effects are highlighted in bold. The coefficients of the factors represent the direction and magnitude of the change in the value of the dependent variable as the given factor changes.

(5) $$y_{1}=1.276+0.295x_{1}+0.141x_{1}^{2}+0.975x_{3}-0.116x_{3}^{2}-0.102x_{1}x_{2}+0.309x_{1}^{2}x_{2}+0.271x_{1}x_{3}+0.117x_{1}^{2}x_{3}-0.089x_{2}^{3}x_{3}+0.120x_{2}^{2}x_{3}^{2}$$

R^2^ = 0.9224, adjR^2^ = 0.8739, MS residual = 0.120.

**Figure 4:**
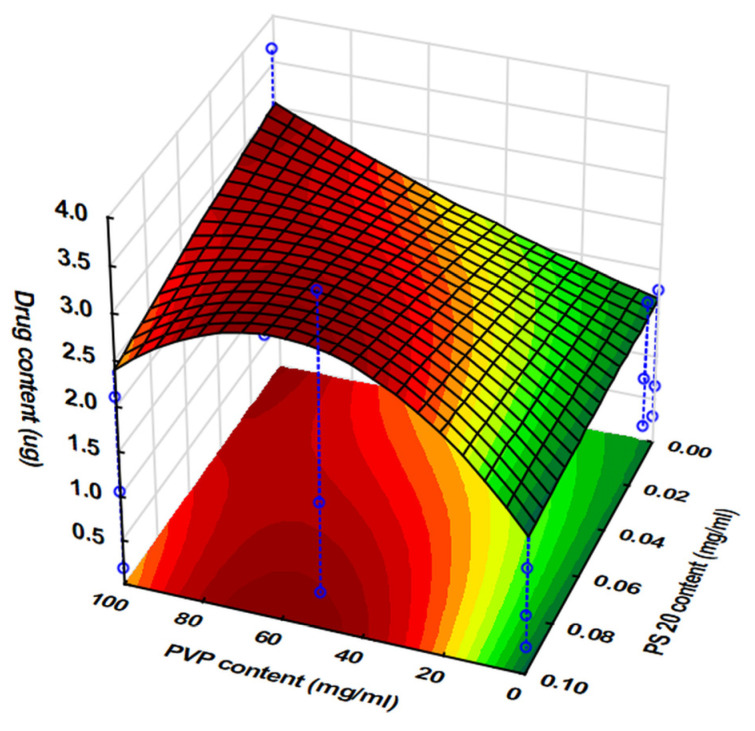
Variation in API content as a function of PVP and PS content at a fixed drop rate (90 drops/point).

The fit of the second-degree polynomial (R^2^ = 0.9224) was adequate to draw reliable conclusions. When examining the effects of the factors, the positive sign of the coefficients of significant (*p* < 0.05) effect indicates that the drug content increased with increasing levels of both the drop number applied per point (*x*_3_) and PVP content (*x*_1_), while the PS content (*x*_2_) had a neglectable effect on this parameter. The increasing PVP content, and hence increasing viscosity, increased the drug content through the increment of the droplet volume. This increment was found to be linear at low PS levels, where the high surface tension of the ink further improved the reachable drop volume (Figure 4). However, as is indicated by the significant (*p* < 0.05) interaction between the exponential component of the PVP content and the linear component of the PS content, the size and volume of droplets decreased due to the decreasing surface tension (Figure 3), and interestingly, this effect was bigger at higher viscosity values (Figure 4), which may have been due in that case to the surface tension not withstanding the increasing weight of the liquid as it accelerated the dripping and thus decreased the available drug content.

**Figure 5:**
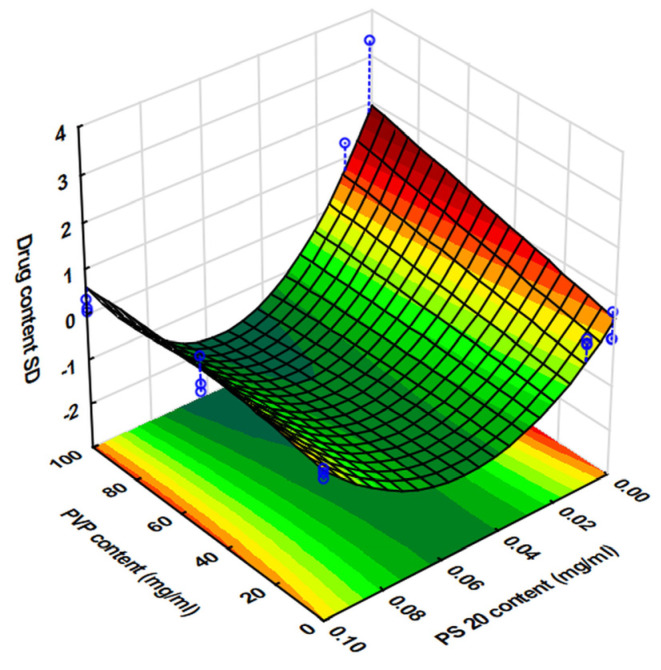
The standard deviation of the API content as a function of PVP and PS content at a fixed drop number (90 drops/point).

Nevertheless, it is clearly visible from the data (Table 6) that the increment of drug content was not in direct proportion with the increment of drop number, which may be due to inaccuracies in the working of the printhead. When examining the dosing accuracy through the analysis of the standard deviation of the drug content, a rather poor model fit was observed in the statistical evaluation Equation (6). The value of R^2^ (adj R^2^ = 0.0942) computed by the software is very low. This may be because the statistical software approximates the nonlinear effects with a parabolic function, which cannot cover the strong nonlinear effect of the PS content due to its logarithmic scaling (Figure 5).

(6) $$\begin{array}{l} y_{2}=0.372+0.168x_{1}-0.128x_{2}+0.209x_{3}-0.288x_{1}x_{2}-0.157x_{1}x_{2}^{2}\\ \;+0.212x_{1}^{2}x_{2}+0.185x_{1}x_{3}-0.173x_{2}x_{3}+0.143x_{2}x_{3}^{2}\\ \;-0.242x_{2}^{2}x_{3}+0.247x_{2}^{2}x_{3}^{2} \end{array}$$

R^2^ = 0.6302, adjR^2^ = 0.3590, MS Residual: 0.2738.

Due to the poor fit, no factors were found with significant effect, and no detailed analysis was carried out due to the lack of a suitable model. However, in general, the increasing PVP content considerably increased the variance in terms of the dosing accuracy due to the corresponding increasing viscosity, which could be effectively compensated by increasing the PS content and thus decreasing the surface tension, and this is well supported by the fact that the highest deviation in the results was observed in the case of ink 7, where—probably due to high viscosity and surface tension—the droplet formation was not synchronous with the trigger signal such that less droplets were dispensed than expected. This may have further caused partial clogging of the nozzle, which is supported by the fact that for ink 7, only a 6-fold increment was observed in the drug content when the drop number was increased from 10 to 90.

### 3.4. Drying Time

In addition to the drug content and dose accuracy, one of the key issues to introduce printing technologies into pharmaceutical manufacturing is the length of the process, which has a major impact on cost-effectiveness. Therefore, the time required for ink delivery and drying of the finished dosage form was measured. In all cases, the print cycles fell within the range of 14 to 77 s depending on the number of drops set. However, this printing time can be effectively reduced by using several nozzles in combination.

**Figure 6:**
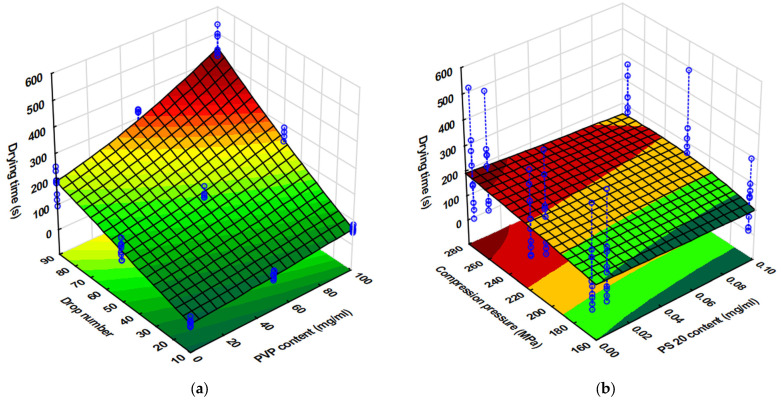
Drying time (s) of inks on porous tablet surface at (**a**) varying droplet counts and PVP contents and (**b**) at varying applied compression forces and PS contents.

The variation in the drying time of the inks was analyzed using the relationship obtained from the 3^4^ full factorial experimental designs Equation (7). The drying times obtained in each experiment are shown in Figure 6.

(7) $$\begin{array}{l} x_{3}=161.4+67.9x_{1}-7.0x_{1}^{2}-8.5x_{2}+125.2x_{3}+27.6x_{4}+6.0x_{4}^{2}-7.4x_{1}x_{2}\\ \;+50.3x_{1}x_{3}-6.5x_{2}x_{3}-5.6x_{1}x_{3}^{2}-5.6x_{1}^{2}x_{3}-6.5x_{2}x_{3}-5.9x_{2}x_{4}\\ \;+4.7x_{2}x_{4}^{2}-4.6x_{2}^{2}x_{4}\pm 18.2x_{3}x_{4}+4.4x_{3}x_{4}^{2} \end{array}$$

R^2^= 0.9670, adjR^2^= 0.9588, MS Residual:694.85.

The fit of the model was adequate in this case to carry out the detailed analysis. It can be clearly seen that the linear effect of all the factors was found to be significant on this CQA, but only the PVP content exerted significant effect on the quadratic level. Similarly to the drug content, the number of printed droplets exerted the highest effect on the drying time both as a single factor and in first-order interaction with the PVP content and the applied compression force. This could obviously have been due to the increasing amount of liquid administered. The higher PVP content further increases the volume of droplets due to higher viscosity, which has an additional effect and increases the exponential increase in drying time at high drop numbers. The increased compression force increases the drying time through the prevention of liquid penetration into the substrate due to decreased porosity. The only factor that had a reducing effect on the drying time was the PS content used to reduce the surface tension, as it facilitated solvent evaporation by reducing the air/liquid interface tension and facilitating the ink’s penetration into the substrate.

### 3.5. Printing Pattern

As a final parameter, the accuracy of the pattern obtained at the end of the printing and drying process was examined, as this can have a significant impact on the readability of individual identification codes if the dosing and labeling of a personalized medicine are aimed with the aid of, for example, a QR code [40]. The result of printing after drying of the inks at 90 drops/point is shown in Table 7.

**Figure 7:**
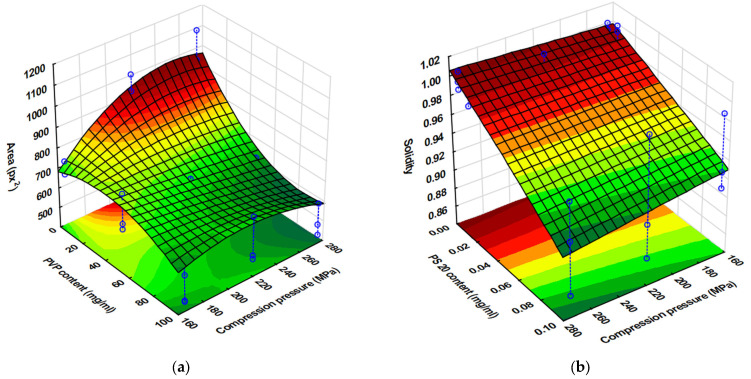
Area (px^2^) of the printed spot (**a**) at varying compression pressures and PVP contents and solidity of the spots (**b**) at varying compression pressures and PS contents.

The complex effect of the porosity of the substrates and the properties of the ink can be seen, and the results are well supported by the results of the spot quantification. The effect of the parameters on the spot size can be seen in Equation (8) and Figure 7a.

(8) $$\begin{array}{l} y_{4}=686.70-163.23x_{1}-32.47x_{1}^{2}+44.26x_{2}+44.83x_{4}+33.09x_{4}^{2}\\ \;+29.5x_{1}x_{2}-15.35x_{1}x_{2}^{2}-69.17x_{1}x_{4}-11.92x_{1}x_{4}^{2}\\ \;-51.5x_{1}^{2}x_{4}-19.58x_{1}^{2}x_{4}^{2}+13.7x_{2}x_{4}^{2}+17.13x_{2}^{2}x_{4} \end{array}$$

R^2^= 0.9687, adjR^2^= 0.9374, MS Residual: 1976.08.

It is clearly visible that the PVP content exerted the highest influence on the spot size, followed by the compression pressure. The interaction of these two parameters was also found to be significant. At low compression pressures, a minor change could be detected in the spot size if the PVP content and so the viscosity of the inks decreased, which may be due the higher porosity and rougher surface promoting ink permeation into the substrate instead of spreading on the surface of substrates. In contrast, at a high compression pressure, the increment in the spot size was exponential.

Regarding pattern accuracy, the effect of the parameters on spot solidity is displayed in Equation (9).

(9) $$\begin{array}{l} y_{5}=0.961-0.013x_{1}-0.018x_{1}^{2}-0.032x_{2}-0.007x_{4}-0.019x_{1}x_{2}\\ \;-0.016x_{1}^{2}x_{2}+0.004x_{1}x_{4}-0.007x_{2}x_{4}+0.003x_{2}^{2}x_{4}^{2} \end{array}$$

R^2^ = 0.9367 adjR^2^ = 0.9031, MS Residual: 0.00016.

The highest coefficient was associated with PS content, and the irregular pattern formed at lower surface free energies (1 mg/mL PS content) can be observed for all three PVP concentrations (Table 7). The low precision due the irregular shape and decreasing solidity (Figure 7b) can be attributed to increased spreading and wetting of the tablet. The spread-enhancing effect of the decreasing substrate porosity at increasing compression pressures is also clearly visible (Figure 7b) and is most noticeable in the case of PVP-free inks due to prolonged drying. Nevertheless, the fact that the solidity decreased less considerably at a low applied compression pressure also supports that higher tablet porosity promotes the penetration of inks into the substrate and their spreading on the surface. The sharp contours at the edges of the pattern were most considerable at a polymer content of 10%. This phenomenon is the so-called “coffee ring effect”, which is caused by the continuous lateral flow of dispersed particles due to the solvent evaporation dominating at the top of the droplet, that eliminates concentration changes [48], but the color differences between the center and edge of the spot might indicate lateral migration and simultaneous “chromatographic separation” of the components. The inhomogeneity caused by this effect is a problem to be eliminated in terms of proper drug distribution and the dispersibility of the printed dosage form, but it can most likely be eliminated by accelerating the drying process or by increasing the ink penetration into the substrate.

## 4. Conclusions

The concept of personalized therapy includes not only the choice of the right API but also the correct adjustment of the therapeutic dose. As a promising option for both the production of personalized therapeutics and simultaneous labeling for identification or anti-counterfeiting purposes, experiments were carried out using inkjet printers. The variation and accuracy of the amount of ink applied to the surface of the porous tablets and the precision of dosing the pattern formed were investigated as functions of the composition of the inks used and the compression force used for preparation of the substrates, which resulted in different porosities.

There are complex interrelations between the ink parameters and properties of porous substrates, which allow for multiple ways for tailoring individualized delivery systems. The incrementation of viscosity has a positive effect on drug content (may improve printing speed) and pattern accuracy but a negative effect on dosing accuracy (due to nozzle clogging) and drying time. The results revealed that viscosity plays a considerably higher role in ink behavior than surface tension.

The decrease in surface tension improves the dosing accuracy due to more precise synchronization of the drop formation and trigger signals and by the prevention of nozzle clogging, and it also improves drying time due to faster evaporation but decreases the drop size and pattern accuracy, since it enables faster penetration of the ink into the substrate, which may cause unbalanced lateral movement of the ink according to the pore texture. Decreasing the substrate porosity increases the drying time and decreases the pattern accuracy.

Higher doses may require the application of delays between the printing of layers or the application of forced drying conditions. Considering the qualitative analysis, the results revealed that the best printing pattern may be achieved with highly viscous and high surface tension inks, but an effort should be made to force the penetration of the ink into the pores of the substrate to prevent the coffee ring effect.

Therefore, it is necessary to further investigate the applicability of the technology with solutions of inks with high viscosity and low surface tension, including the API.

## Figures and Tables

**Table 1:** Composition of inks.

Ink No.	Kollidon 25 (PVP) (mg/mL)	Polysorbate 20 (PS) (mg/mL)
1	0	0.01
2		0.1
3		1
4	50	0.01
5		0.1
6		1
7	100	0.01
8		0.1
9		1

**Table 2:** System specific independent (x) and dependent (y) variables.

	Factor (x)	x1	x2	x3	x4
CQA (y)		PVP Content (m/m %)	PS-Content (mg/mL)	Drop Number/Pixel	Compression Pressure (MPa)
y_1_ API content		x	x	x	
y_2_ dosing accuracy		x	x	x	
y_3_ drying time		x	x	x	x
y_4_ spot area		x	x		x
y_5_ spot solidity		x	x		x

**Table 3:** The values of the independent variables recorded at certain levels (PS content is interpreted on a logarithmic scale).

Factor	−1 Level	0 Level	+1 Level
x_1_	0	50	100
x_2_	0.01	0.1	1
x_3_	10	50	90
x_4_	165	220	275

**Table 4:** Properties of tablet substrates.

Compression Pressure (MPa)	Height (mm)	Diameter (mm)	Mass (g)	Apparent Density (g/cm^3^)	True Density (g/cm^3^)	Porosity (%)
165	1.207 ± 0.087	13.019 ± 0.020	0.2147 ± 0.016	1.336 ± 0.005	1.241 ± 0.001	22.78 ± 0.33
220	1.147 ± 0.025	13.009 ± 0.012	0.2114 ± 0.006	1.386 ± 0.025	1.230 ± 0.001	19.38 ± 1.45
275	1.161 ± 0.131	13.012 ± 0.006	0.2035 ± 0.034	1.313 ± 0.125	1.236 ± 0.002	17.93 ± 2.18

**Table 5:** Surface tension of inks (25 °C).

Ink No.	Average Surface Tension (mN/m)	SD
1	33.7681	0.5178
2	32.2044	0.6705
3	31.2788	0.2357
4	56.0471	1.1431
5	39.5393	0.0413
6	30.8792	0.1735
7	38.2078	0.9537
8	30.3892	0.4115
9	29.4757	0.0735

**Table 6:** The amount of Brilliant Blue dye printed on the tablets.

	Average Dye Content ± SD (µg)	Drop Number/Pixel					
Sample Number of the Ink		10		50		90	
		Average	±SD	Average	±SD	Average	±SD
1		0.332	0.072	0.687	0.083	1.78	0.671
2		0.431	0.209	0.993	0.243	1.853	0.328
3		0.306	0.093	0.663	0.205	1.19	0.298
4		0.295	0.279	1.068	0.064	2.137	0.064
5		0.245	0.183	0.993	0.223	1.94	0.149
6		0.416	0.078	1.412	0.246	3.671	0.865
7		0.6	0.48	1.093	0.36	3.671	3.359
8		0.602	0.261	2.125	1.352	2.625	0.336
9		0.183	0.127	1.057	0.056	2.1	0.32

**Table 7:** Print pattern on the surface of the tablets (90 drop/px).

PVP (mg/mL)		0			50			100		
PS (mg/mL)		0.01	0.1	1	0.01	0.1	1	0.01	0.1	1
Compression force (t)	3	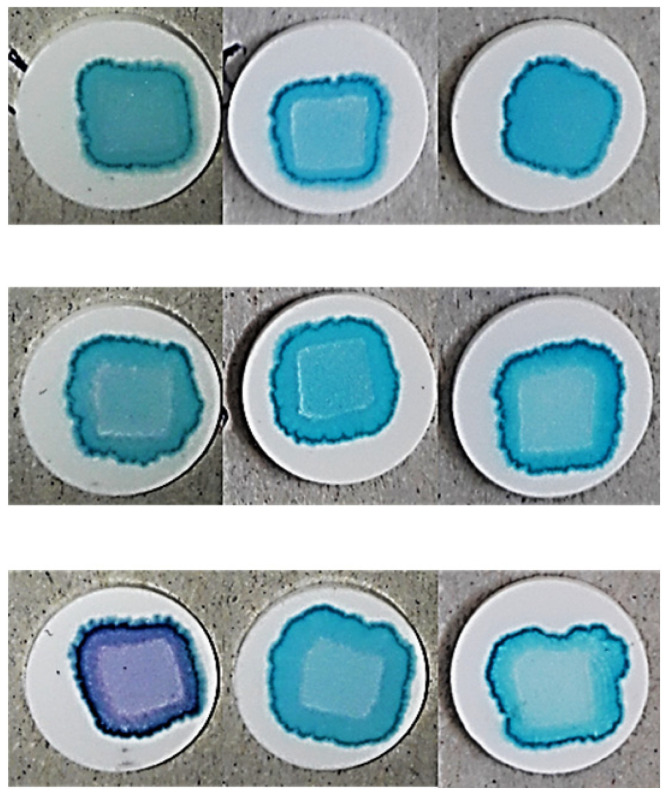			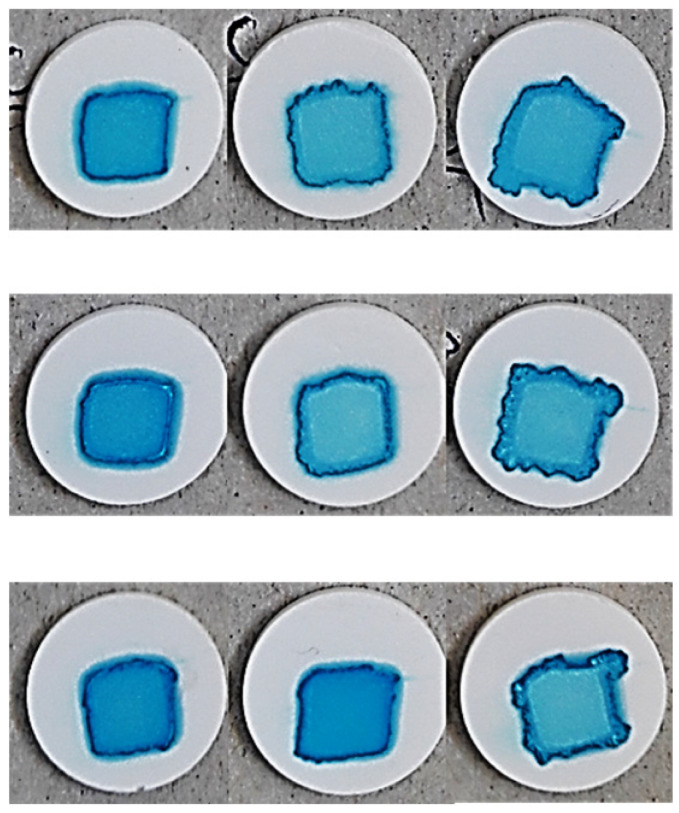			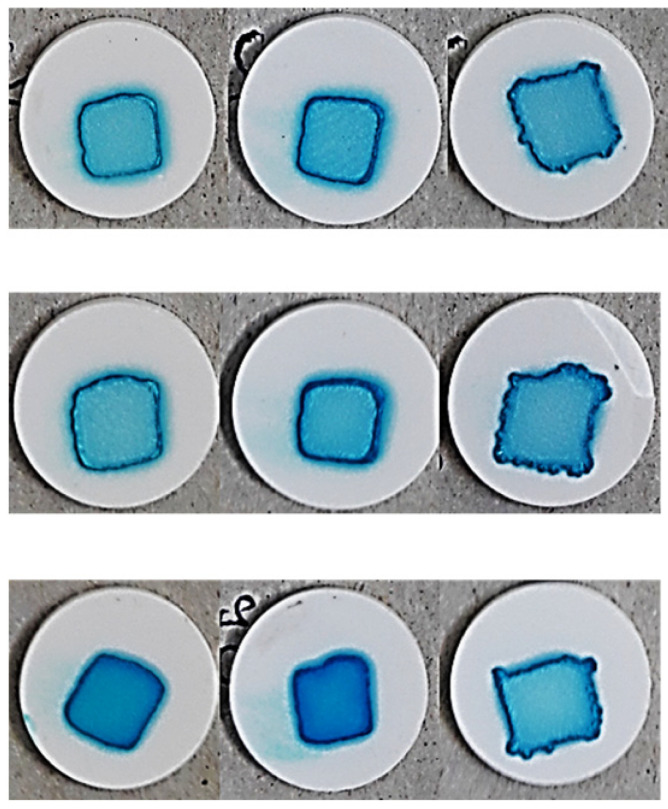		
	4									
	5									

## Data Availability

The data are available upon request.

## Supplementary Materials

The following supporting information can be downloaded at https://www.mdpi.com/article/10.3390/pharmaceutics17070908/s1, Description of DoE methodology.

## Abbreviations

The following abbreviations are used in this manuscript:

CQA	Critical quality attribute
DoD	Drop-on-demand
IJP	Inkjet printing
PVP	Polyvinylpyrrolidone
PS	Polysorbate
QbD	Quality by Design
