# Supplementary material for: Behavior of Aqueous Medicated Inks on Porous Tablet Surfaces
Source: Pharmaceutics. 2025 Jul 14;17(7):908. doi: 10.3390/pharmaceutics17070908 (PMC12298566; doi:10.3390/pharmaceutics17070908)
Supplement: Supplementary file 1 [file pharmaceutics-17-00908-s001.zip › pharmaceutics-3611378-supplementary.pdf]

Supplementary material – Description of the Design of Experiments (DoE) and Response Surface Methodology (RSM)

Design of Experiments (aka Factorial design or Response Surface Methodology) is a commonly used statistical technique to minimize the number of necessary experiments and maximize the gathered information on the relationships between independent and dependent variables. Independent variables (factors) could be Critical Material Attributes (CMAs) of materials used in the formulations, Critical Process Parameters (CPPs) or Critical Environmental Parameters (CEPs), which as per definition are variables that can be measured precisely, can be varied, but have a fixed value in time, and are directly related to the subject of the study. Factors should be measured with the greatest possible accuracy (this determines the minimum distance between the possible factor levels), controllable (should be kept constant during the experiment), and independent from each other (all factors should be set to the given value, independently from the actual value of the other factors). The selection of factor levels can be based on the results of previous experience, literature data or preliminary studies. Base (0) level is usually a commonly used value of the given factor. The 0 level should be an arithmetic mean of the minimum (-1) and maximum (+1) values, which enables the correct interpretation of the results.

The subject of the study, or dependent variables (optimization parameters) are usually the Critical Quality Attributes (CQAs) of the products (such as tablet hardness, drug content, release rate etc.) Among the investigated CQAs one could be selected as the optimization parameter, the other CQAs can serve as constrainers.

DoE is based on the simultaneous variation of the factors which enables the estimation of the individual effect of the various factors and the effect of their interactions on the optimization parameters, thus providing fast and complex information about the system under study. Full factorial design is the type where all combinations of the possible factor levels are presented. For two factors and 3 levels it can be described with the following correlation matrix:

| **x1** | **x2** |
|---|---|
| -1 | -1 |
| 0 | -1 |
| +1 | -1 |
| -1 | 0 |
| 0 | 0 |
| +1 | 0 |
| -1 | +1 |
| 0 | +1 |
| +1 | +1 |

The number of necessary experiments equals with $3^n$, where n is the number of factors involved in the study.

For the two factor case, the result of the experiments can be described with the following general equation:

$$y=b_0+b_1x_1+b_{11}x_1^2+b_2x_2+b_{22}x_2^2+b_{12}x_1x_2+b_{112}x_1^2x_2+b_{122}x_1x_2^2+b_{1122}x_1^2x_2^2$$

where y is the studied CQA, $x_1$ and $x_{n2}$ are the factors involved in the study, $b_0$ is the overall mean of the experimental results and $b_1$ and $b_2$ are coefficients which shows the change of the response if the corresponding factor is changed from the 0 to the +1 level. The values of the coefficients are calculated as the weighted mean of the results, where the weighting factor is the actual level of the given factor.

The statistical evaluation of the equation is based on the hypothesis significance of the factor effects is based on ANOVA study, where the compared populations are the results of those cases where the investigated factor is at -1, 0 or +1 level. The obtained model is then fitted to the experimental data by a conventional curve fitting algorithm of the software, which provides the coefficient of determination ($R^2$) and calculates the MS Residual for those points which are not completely on the fitted model surface. As $R^2$ value depends on the number of determinants in the equation adj $R^2$ is also calculated which is independent from the number of applied determinants in the model. The unnecessary determinants to be neglected from the equation of the response surface can be selected through the maximization of the adj $R^2$ value. and minimizing the MS residual, and so the goodness of fit can be improved.
